# Estimating the release of inflammatory factors and use of glucocorticoid therapy for COVID-19 patients with comorbidities

**DOI:** 10.18632/aging.202172

**Published:** 2020-11-24

**Authors:** Dan Han, Chunfen Peng, Rui Meng, Jing Yao, Qiong Zhou, Yong Xiao, Hong Ma

**Affiliations:** 1 Cancer Center, Union Hospital, Tongji Medical College, Huazhong University of Science and Technology, Wuhan, Hubei, China; 2 Department of Gastrointestinal Surgery, Union Hospital, Tongji Medical College, Huazhong University of Science and Technology, Wuhan, Hubei, China; 3 Department of Respiratory and Critical Care Medicine, Union Hospital, Tongji Medical College, Huazhong University of Science and Technology, Wuhan, China

**Keywords:** COVID-19, comorbidities, immunological characteristics, inflammatory cytokines, glucocorticoid therapy

## Abstract

COVID-19 exhibits both variability and rapid progression, particularly in patients with comorbidities such as diabetes, hypertension or cancer. To determine how these underlying disorders exacerbate pneumonia in COVID-19, we evaluated 79 patients with severe COVID-19 and grouped them according to whether or not they had comorbidities. Clinical information, laboratory examinations, immunological function, and treatment outcomes were retrospectively analyzed. Our study revealed that severe COVID-19 patients with comorbidities had higher levels of inflammatory indices, including blood interferon-γ, interleukin (IL)-6 and c-reactive protein levels as well as the erythrocyte sedimentation rate. These were accompanied by lymphopenia, hypokalemia, hypoalbuminemia, a decrease in either CD4+ T cells or lymphocyte count, and coagulation disorders, which were closely related to poor prognosis. Patients with comorbidities also had longer disease remission times (27 ± 6.7 days) than those without comorbidities (20 ± 6.5 days). Cox multivariate analysis indicated that glucocorticoid therapy and IL-6 were independent prognostic factors. Our findings suggest that coexisting comorbidities aggravate COVID-19 through the excessive release of inflammatory factors and that glucocorticoid therapy may be beneficial.

## INTRODUCTION

Coronaviruses (CoVs) exist in human and animal hosts, causing severe respiratory tract and intestinal infections [1, 2]. In December 2019, an unknown pneumonia was reported in Wuhan, Hubei Province, China and identified as the seventh highly pathogenic new coronavirus infecting humans since the Severe Acute Respiratory Syndrome CoV (SARS-CoV) in 2003 and Middle East Respiratory Syndrome CoV (MERS-CoV) in 2012 [3, 4]. Considering its global threat, the novel CoV was named SARS-CoV2, and the disease caused by it was named coronavirus disease 2019 (COVID-19) by the World Health Organization (WHO) on February 11, 2020 [4].

COVID-19 is characterized by high mortality rates, high infectivity, and strong pathogenicity. Patients with mild COVID-19 may have fever, dry cough, and fatigue, recovering in a short time while severe patients manifest rapidly progressive acute respiratory distress syndrome (ARDS), with severe metabolic acidosis and coagulation dysfunction [5, 6]. COVID-19 diagnosis depends on the detection of RNA virus nucleic acid from respiratory samples and chest computed tomography (CT) images characterized as multiple, ground-glass opacities and infiltrates in bilateral lungs. Clinical laboratory results and assessments of immune function, including serum interleukin (IL)-6, IL-10, tumor necrosis factor-α (TNF-α) and interferon-γ (IFN-γ) levels, as well as T-lymphocyte CD4+ and CD8+ cell counts, also influence treatment outcomes [7]. In patients with underlying comorbidities such as diabetes, hypertension, or cancer; COVID-19 has the characteristics of variability and rapid progress [3, 4]. Our study examined how these underlying disorders exacerbated pneumonia in COVID-19.

Our cancer center is affiliated with the Union Hospital (Hubei, China) and located in Wuhan, where COVID-19 was first reported. Severe COVID-19 patients were admitted to our cancer center from February 10 to March 22, 2020. Our research retrospectively analyzed the basic clinical characteristics, immune state, and radiological manifestations in these severe COVID-19 patients. We also explored the internal cause for the effects of coexisting comorbidities on COVID-19 prognosis to provide more treatment strategies.

## RESULTS

### Patient baseline characteristics

A total of 79 eligible cases were enrolled in this study and retrospective analysis was performed. All of the cases met the criteria for severe COVID-19 according to the guidelines for diagnosis and treatment issued by the National Health Commission of the People’s Republic of China. Among them, 45 cases had comorbidities, including malignancy in 11 (24.5%), diabetes mellitus in 10 (22.2%) and hypertension in 24 (53.3%). The age of patients with comorbidities was significantly higher than those without comorbidities, and the median age in the two groups were 69 and 56 years, respectively (*P* < 0.01). The percentage of male subjects in the comorbidity and no comorbidities groups were 53.3% and 41.1%, respectively, but did not statistically differ. The clinical presentations of patients at admission were fever (74.6%), dry cough (64.5%), and dyspnea (56.9%), with no significant difference between the comorbidity and non-comorbidity groups. All enrolled patients received antiviral treatment. The patients received antibacterial treatment at a rate of 86.7% (comorbidities group) versus 73.5% (no comorbidities group, *P* = 0.14). 86.6% of patients in the comorbidities group received nutritional supports, which was significantly higher than that of the non-comorbidities group (61.7%, Table 1).

**Table 1:** Clinical baseline characteristics of severe COVID-19 with and without comorbidities.

**Patient characteristics**	**ALL patients**	**None comorbidities**	**Comorbidities**	**P value**
	**(n=79)**	**(n=34)**	**(n=45)**	
Age, median yrs	65 (28.0-96.0)	56 (28.0-83.0)	69 (37.0-96.0)	0.000*
> 60	47/79 (59.4%)	18/34 (52.9%)	39/45 (86.6%)	0.002*
Males, n (%)	38/79 (48.1%)	14/34 (41.1%)	24/45 (53.3%)	0.290
Any comorbidity, n/N (%)	45/79 (56.9%)	-	45/45 (100.0%)	NA
Hypertension, n/N (%)	24/79 (30.3%)	-	24/45 (53.3%)	NA
Diabetes, n/N (%)	10/79 (12.7%)	-	10/45 (22.2%)	NA
Cancer, n/N (%)	11/79 (13.9%)	-	11/45 (24.5%)	NA
Signs and symptoms				
Fever, n/N (%)	59/79 (74.6%)	26/34 (76.4%)	33/45 (73.3%)	0.755
Highest temperature, ° C	38.4 (37.3-40.0)	38.3 (37.3-39.3)	38.6 (37.5-40.0)	0.148
38.1-39.0° C, n/N (%)	39/59 (66.1%)	14/26 (53.8%)	25/33 (75.7%)	0.409
>39.0° C, n/N (%)	13/59 (22.0%)	4/26 (15.3%)	9/33 (27.2%)	0.282
Cough, n/N (%)	51/79 (64.5%)	23/34 (67.6%)	28/45 (62.2%)	0.623
Sputum production, n/N (%)	16/79 (20.2%)	6/34 (44.1%)	10/45 (22.2%)	0.319
Myalgia, n/N (%)	28/79 (35.4%)	15/34 (81.0%)	13/45 (28.8%)	0.099
Dyspnea, n/N (%)	45/79 (56.9%)	19/34 (42.2%)	26/45 (57.7%)	0.868
Sore throat, n/N (%)	10/79 (12.6%)	6/34 (17.6%)	24/45 (53.3%)	0.252
Diarrhea, n/N (%)	16/79 (20.2%)	9/34 (26.4%)	7/45 (15.5%)	0.237
Nausea and vomiting, n/N (%)	18/79 (22.7%)	10/34 (29.4%)	8/45 (17.7%)	0.228
Headache, n/N (%)	14/79 (17.7%)	6/34 (17.6%)	8/45 (17.7%)	0.988
Systolic pressure, mm Hg	129.7 (98.0-192.0)	124.5 (107.0-151.0)	133.6 (98.0-192.0)	0.013*
>140mmHg, n/N (%)	17/79 (21.5%)	3/34 (8.8%)	14/45 (31.1%)	0.011*
Heart rate, bpm	86.9 (56.0-134.0)	86.3 (58.0-131.0)	87.4 (56.0-134.0)	0.759
> 80bpm, n/N (%)	35/79 (44.3%)	15/34 (44.1%)	20/45 (44.4%)	0.977
Treatment option				
Anti-virus therapy, n/N (%)	79/79 (100.0%)	34/34 (100.0%)	45/45 (100.0%)	NA
Antibacterial therapy, n/N (%)	64/79 (81.0%)	25/34 (73.5%)	39/45 (86.7%)	0.144
Supportive treatment, n/N (%)	59/79 (74.6%)	21/34 (61.7%)	38/45 (86.6%)	0.022*
Glucocorticoid therapy, n/N (%)	29/79 (36.7%)	6/34 (17.6%)	23/45 (51.1%)	0.003*

Abbreviation: APTT, Activated partial thromboplastin time; COVID-19, coronavirus disease 2019; ESR, Erythrocyte Sedimentation Rate; High-sensitivity C-reactive protein, HS-CRP; N, number of total samples in each group; n, positive case number in each group. Data are expressed as mean (interquartile ranges, IQR) and n/N (%). P values mean the comparison between severe COVID-19 with or without comorbidities, *p < 0.05 statistically significant.

### Routine laboratory tests and CT features analysis

In baseline laboratory tests, most of patients showed abnormal white cell count (normal or mildly low), while patients in comorbidities group showed obvious lymphopenia (57.7%) and thrombocytopenia (22.2%) and a relative increase in the number of granulocytes (26.6%). Additionally, obvious malnutrition and abnormal coagulation, such as hypokalemia accounting for 22.2%, hypoproteinemia (26.6%), and marked rise in D-dimer and Fibrinogen were observed in comorbidities group. Hepatic dysfunction was also common in severe COVID-19 patients, showed with increased serum Aspartate aminotransferase (AST) and Alanine aminotransferase (ALT) levels, but without differences between the groups. Additionally, patients with comorbidities showed prominent increased inflammatory parameters, such as CRP (56.8% vs. 26.4%), ESR (91.1% vs. 82.3%) compared with non-comorbidities. Radiographic findings in COVID-19 patients included clumped, ground-glass and interstitial lesions, involving bilateral lung or multilobar lung, but without differences between two groups (Table 2).

**Table 2:** Results of routine laboratory examination and CT images from severe COVID-19 with and without comorbidities.

	**Normal range**	**ALL patients**	**None comorbidities**	**Comorbidities**	**P value**
		**(n=79)**	**(n=34)**	**(n=45)**	
White blood cell count, × 10^9^/L	3.5-9.5	6.0 (1.7-17.8)	5.5 (2.5-10.1)	6.4 (1.7-17.8)	0.139
< 4, n/N (%)		21/79 (26.5%)	8/34 (23.5%)	13/45 (28.8%)	0.599
≥ 10, n/N (%)		6/79 (7.5%)	1/34 (2.9%)	5/45 (11.1%)	0.147
Neutrophil count, × 10^9^/L	1.8-6.3	4.3 (1.4-16.3)	3.7 (1.4-8.6)	4.9 (1.4-16.4)	0.030*
> 6.3× 10^9^/L, n/N (%)		15/79 (18.9%)	3/34 (8.8%)	12/45 (26.6%)	0.034*
Lymphocyte count, × 10^9^/L	1.1-3.2	1.0 (0.52-1.9)	1.2 (0.5-1.9)	0.9 (0.6-1.8)	0.029*
< 0.9, n/N (%)		38/79 (48.1%)	12/34 (35.2%)	26/45 (57.7%)	0.048*
Hemoglobin, g/L	130.0-175.0	121.0 (75.0-156.0)	124.3 (91.0-154.0)	118.5 (75.0-156.0)	0.104
< 110g/L, n/N (%)		12/79 (15.1%)	2/34 (5.8%)	10/45 (22.2%)	0.037*
Platelet count, × 10^9^/L	125.0-350.0	224.5 (62.0-437.0)	229.4 (102.0-437.0)	220.9 (62.0-422.0)	0.665
< 120× 10^9^/L, n/N (%)		12/79 (15.1%)	2/34 (5.8%)	10/45 (22.2%)	0.032*
Alanine aminotransferase, U/L	≤ 41.0	41.3 (10.0-434.0)	29.2 (10.0-241.0)	50.2 (10.0-434.0)	0.047*
> 50U/L, n/N (%)		17/79 (21.5%)	4/34 (11.7%)	13/45 (28.8%)	0.019*
Aspartate aminotransferase, U/L	≤ 40.0	39.8 (11.0-639.0)	27.1 (15.0-196.0)	49.1 (11.0-639.0)	0.127
> 50U/L, n/N (%)		11/79 (13.9%)	2/34 (5.8%)	9/45 (20%)	0.062
Total bilirubin, mmol/L	≤ 26.0	13.8 (4.9-65.3)	12.6 (4.9-21.5)	14.8 (5.3-65.3)	0.183
< 32 mmol/L, n/N (%)		3/79 (3.7%)	0/34 (0%)	3/45 (6.6%)	0.128
Albumin, g/L	35.0-52.0	34.6 (28.0-46.5)	37.2 (29.4-46.5)	32.6 (28.0-42.4)	0.000*
< 32 g/L, n/N (%)		16/79 (20.2%)	4/34 (11.7%)	12/45 (26.6%)	0.003*
Sensitive troponin, ng/ml	< 0.1	6.6 (0.5-43.7)	4.6 (0.5-22.0)	8.0 (0.7-43.7)	0.076
> 26 ng/ml, n/N (%)		5/79 (6.3%)	0/34 (0%)	5/45 (11.1%)	0.045*
Lactate dehydrogenase, U/L	135.0-225.0	245.4 (80.0-544.0)	218.6 (80.0-517.0)	265.3 (135.0-544.0)	0.016*
> 225 U/L, n/N (%)		37/79 (46.8%)	11/34 (32.3%)	26/45 (57.7%)	0.015*
Potassium, mmol /L	3.5-5.5	3.7 (2.9-4.5)	3.8 (3.2-4.5)	3.6 (2.9-4.3)	0.001*
< 3.3mmol/L, n/N (%)		12/79 (15.1%)	2/34 (5.8%)	10/45 (22.2%)	0.029*
Sodium, mmol/L	135-145	138.6 (132.8-148.0)	138.2 (132.8-144)	139.0 (133.0-148.0)	0.849
< 135mmol/L, n/N (%)		6/79 (7.5%)	0/34 (0%)	6/45 (13.3%)	0.027*
Prothrombin time, seconds	11.5-14.5	13.4 (11.9-16.6)	13.2 (11.9-16.6)	13.6 (12.2-15.4)	0.066
> 14.5 seconds, n/N (%)		8/79 (10.1%)	2/34 (5.8%)	6/45 (13.3%)	0.259
APTT, seconds	29.0-42.0	37.3 (30.4-47.3)	37.4 (30.4-45.6)	37.2 (30.4-47.3)	0.838
> 42 seconds, n/N (%)		10/79 (12.6%)	2/34 (5.8%)	8/45 (17.7%)	0.096
D-dimer, μg/mL	< 0.5	2.5 (0.2-34.5)	0.9 (0.2-5.92)	3.6 (0.2-34.5)	0.005*
> 2μg/mL, n/N (%)		22/70 (31.4%)	4/28 (14.2%)	18/42 (42.8%)	0.011*
0.5-2μg/mL, n/N (%)		21/70 (30.0%)	4/28 (14.2%)	17/42 (40.4%)	0.023*
Fibrinogen, g/l	2.0-4.0	4.3 (0.4-8.5)	0.9 (2.1-7.3)	4.4 (0.4-8.5)	0.046*
> 4g/l, n/N (%)		40/79 (50.6%)	11/34 (32.3%)	29/45 (64.4%)	0.004*
CRP, mg/L	< 1.0	38.4 (0.4-246.0)	23.0 (1.3-104.0)	50.3 (0.4-246.0)	0.002*
> 30mg/L, n/N (%)		34/78 (43.5%)	9/34 (26.4%)	25/44 (56.8%)	0.007*
ESR, mm/h	0.0-15.0	53.1 (3.0-202.0)	48.0 (3.0-202.0)	57.5 (6.0-140.0)	0.022*
> 15mm/h, n/N (%)		69/79 (87.3%)	28/34 (82.3%)	41/45 (91.1%)	0.252
CT image					
Bilateral lung infection		71/79 (89.8%)	27/34 (79.4%)	42/45 (93.3%)	0.065

Abbreviation: APTT, Activated partial thromboplastin time; COVID-19, coronavirus disease 2019; ESR, Erythrocyte Sedimentation Rate; High-sensitivity C-reactive protein, HS-CRP; N, number of total samples in each group; n, positive case number in each group. Data are expressed as mean (interquartile ranges, IQR) and n/N (%).

P values mean the comparison between severe COVID-19 with or without comorbidities, *p < 0.05 statistically significant.

### Inflammatory factors and lymphocyte subset analysis

**Figure 1:**
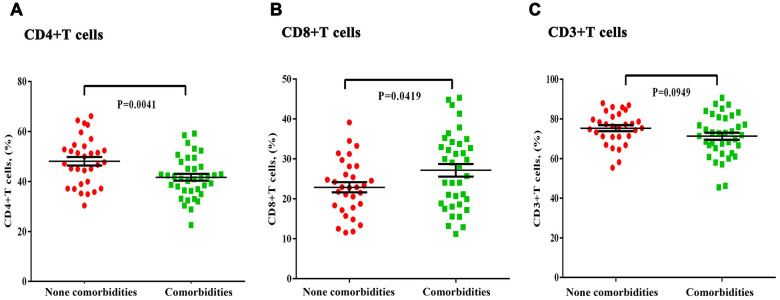
**Differences in the T-lymphocyte subsets in severe COVID-19 patients with and without chronic comorbidities**. (**A**) Significant decreases in the proportion of CD4+T cells was observed in the comorbidities group. (**B**) There was no significant difference in CD8+ or (**C**) CD3+ T cell proportions between the comorbidities and non-comorbidities groups.

**Figure 2:**
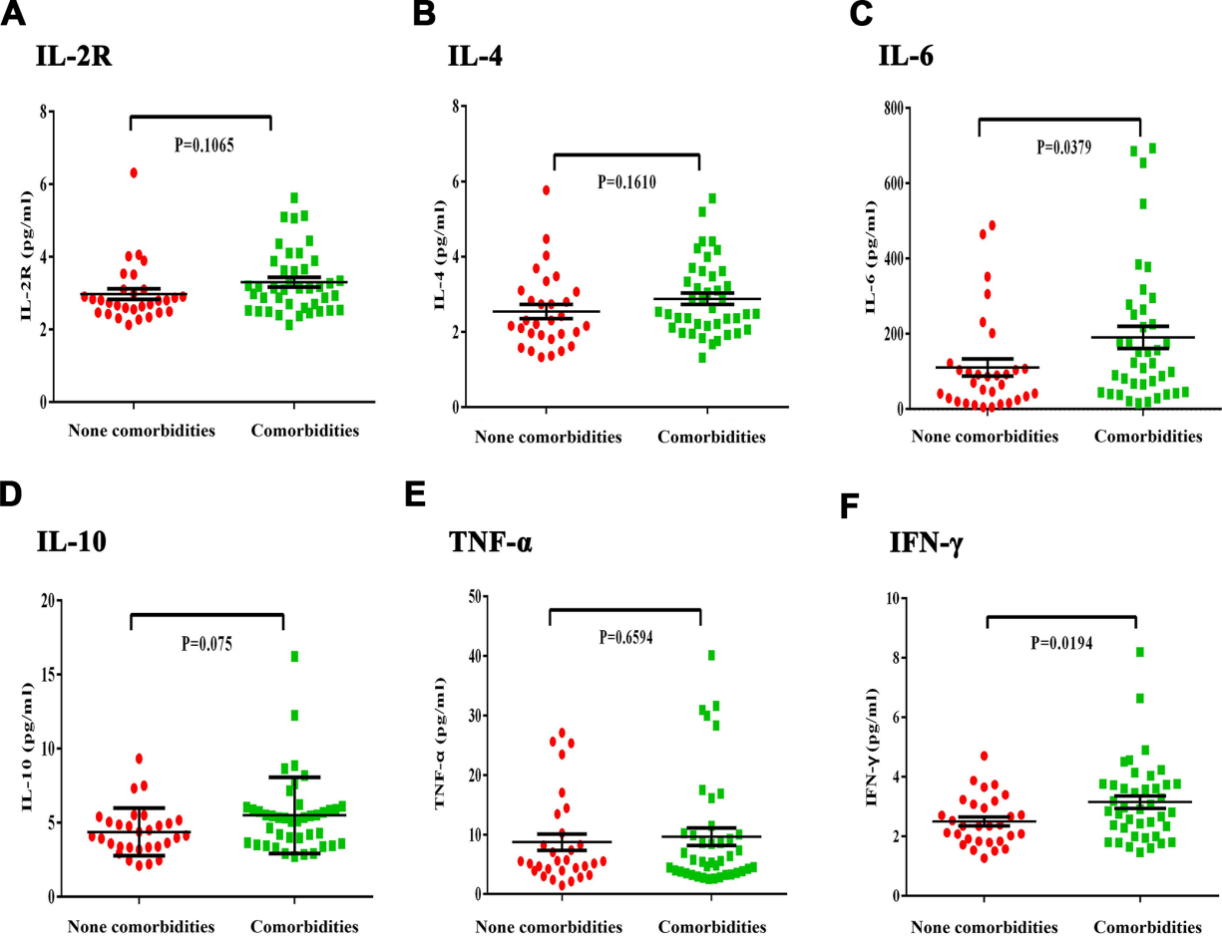
**Differences in inflammatory cytokines between severe COVID-19 patients with and without comorbidities**. (**A**, **B**) There was no obvious difference in IL-2R and IL-4 level between the comorbidities and non-comorbidities groups. (**C**) IL-6 levels were significantly increased in the comorbidities group. (**D**, **E**) There was no obvious difference in IL-10 and TNF-α levels between the comorbidities and non-comorbidities groups. (**F**) The comorbidities group had significantly increased IFN-γ levels.

**Figure 3:**
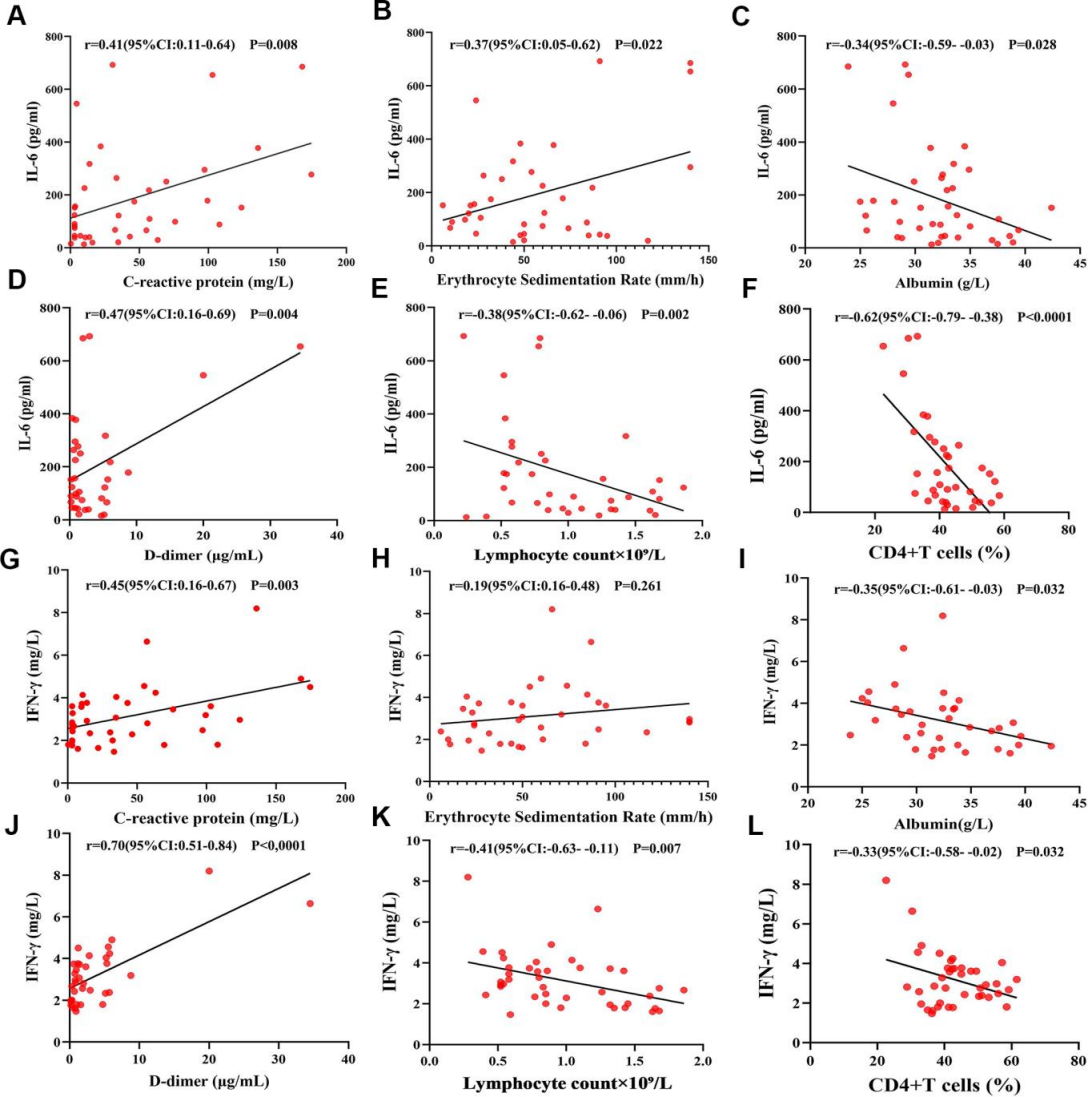
**Correlations between IL-6, IFN-γ and ESR, CRP, albumin, D-dimer, lymphocyte count, and CD4+ T cells.** (**A**, **B**, **D**) IL-6 was positively correlated with C-reactive protein, erythrocyte sedimentation rate and D-dimer. (**C**, **E**, **F**) IL-6 was negatively correlated with albumin, lymphocyte count, and the proportion of CD4+ T cells. (**G**, **J**) IFN-γ was positively correlated with C-reactive protein and D-dimer. (**H**) IFN-γ had no significant correlation with the erythrocyte sedimentation rate. (**I**, **K**, **L**) IFN-γ was negatively correlated with albumin, lymphocyte count the proportion of CD4+ T cells.

The proportion of CD3+, CD4+, and CD8+ T cell subsets in peripheral blood was also determined (Figure 1A–1C). We found that there was a decrease in the proportion of CD4+ T cells in both groups, which was more pronounced in the comorbidities group (Figure 1A). There was a decrease in the proportion of CD4+ T cells in both groups, which was more pronounced in the underlying diseases group. Baseline serum levels of inflammatory factors IL-2R, IL-4, IL-6, IL-10, and IFN-γ were analyzed (Figure 2A–2F). The patients with comorbidities exhibited higher serum levels of IL-6 and IFN-γ when compared to patients without comorbidities (Figure 2C, 2F). Furthermore, elevated levels of IL-6 were positively correlated with an increase in CRP (r=0.41, P=0.008), ESR (r=0.37, P=0.022) and D-dimer (r=0.47, P=0.004) (Figure 3A, 3B, 3D); and negatively correlated with albumin (r=-0.34, P=0.028), lymphocyte counts (r=-0.38, P=0.002), and the proportion of CD4+T cells subsets (r=-0.62, P<0.0001) (Figure 3C, 3E, 3F). A negative correlation existed between IFN-γ and albumin (r=-0.35, P=0.032), lymphocyte counts (r=-0.41, P=0.007), and the proportion of CD4+T cells subsets (r=-0.33, P=0.032) (Figure 3I, 3K, 3L); while a positive correlation existed between CRP (r=0.45, P=0.003) and D-dimer (r=0.70, P<0.0001) as shown in Figure 3G, 3J.

### Treatment outcome and prognosis

**Figure 4:**
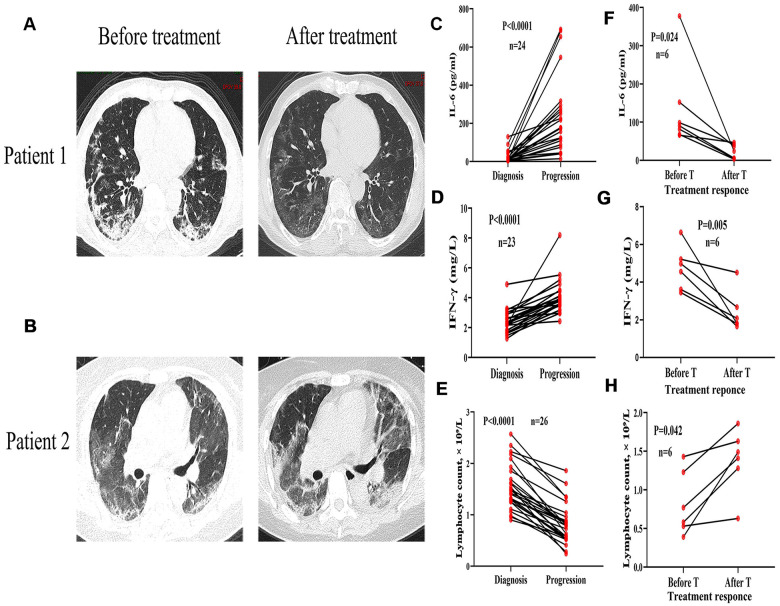
**Variation of chest CT images, inflammatory cytokines, and lymphocyte count in severe COVID-19 patients with and without comorbidities.** CT images from a 70-year-old male with diabetes showing bilateral lungs before treatment and infiltrating inflammation after one week of treatment. (**B**) CT images from a 72-year-old female with diabetes and hypertension showing infiltrating inflammation in bilateral lungs before treatment and deteriorated lung inflammation after one week of treatment. (**C**–**E**) Serum levels of IL-6 and IFN-γ progressively increased and lymphocytes decreased with disease progression. (**F**–**H**) Decreased serum levels of IL-6 and IFN-γ, and recovered lymphocytes before and after glucocorticoid treatment (T).

All patients enrolled in this study were regularly examined for chest CT, blood, and serum levels of inflammatory cytokines every 7-10 days following COVID-19 treatment to evaluate the changes in disease development. The signs of disease remission included: relief of symptoms, negative nucleic acid detection of COVID-19 (twice within a 24-h interval), and if the CT images showed a reduction in the size of pulmonary lesions > 20% (Figure 4A). In the comorbidities group, 30 cases showed progress during treatment as characterized by inflammation development in the CT images (Figure 4B), accompanied by elevated levels of IL-6 and IFN-γ, and decreased lymphocyte counts (Figure 4C–4E). In 23 cases of the comorbidities group, decreased levels of IL-6 and IFN-γ as well as improved lymphocyte counts were observed after glucocorticoid treatment (Figure 4F–4H).

**Figure 5:**
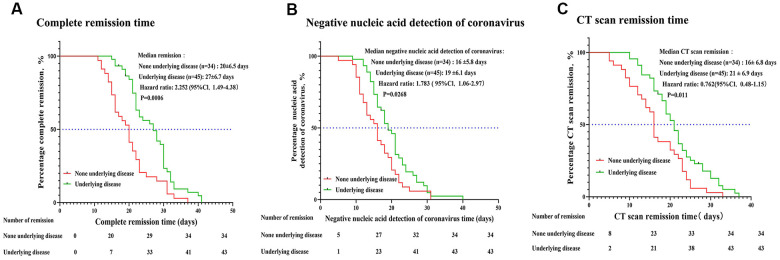
**Disease remission time in severe COVID-19 patients with and without comorbidities.** The time for disease remission was evaluated by the Kaplan-Meier method. The median remission time, hazard ratio (HR) and 95% confidence interval (CI) are shown. (**A**) Complete remission time (time from onset treatment to negative nucleic acid detection of coronavirus combined with CT images absorption). (**B**) Time for negative coronavirus nucleic acid detection (time from onset treatment to negative nucleic acid detection of coronavirus). (**C**) Time for CT images to show disease remission.

The time for complete remission, negative coronavirus nucleic acid detection, and CT images remission (27 ± 6.7, 19 ± 6.1, 21 ± 6.9 days, respectively) in the comorbidities group was significantly longer than that in non-comorbidities group (20 ± 6.5, 16 ± 5.8, 16 ± 6.8 days) (Figure 5A–5C). Multivariate COX regression analysis showed that serum levels of IL-6 and glucocorticoid treatment were independent and protective factors, respectively, for the prognosis of severe COVID-19 patients with coexisting comorbidities (Table 3).

**Table 3:** Cox regression analysis of clinical factors for disease relief time in severe COVID-19 with comorbidities.

	**HR**	**Univariate Analysis**	**P value**	**HR**	**Multivariate Analysis**	**P value**
		**95% CI**			**95% CI**	
Age > 60yrs	0.034	0.306-3.042	0.952			
Male	0.471	0.579-4.429	0.364			
Systolic pressure > 140mmHg	0.692	0.562-7.089	0.285			
Heart rate>80bpm	0.633	0.643-5.518	0.248			
Respiratory rate > 20per min	0.672	0.441-8.693	0.377			
Glucocorticoid therapy	4.103	1.156-14.561	0.029*	14.095	2.339-24.952	0.004*
WBC count ≥ 9.5× 10^9^/L	0.164	0.239-3.011	0.799			
Neutrophil count ≥ 6.3× 10^9^/L	0.274	0.449-3.848	0.618			
Platelet count < 120× 10^9^/L	1.147	0.413-24.0	0.269			
Lymphocyte count < 0.9× 10^9^/L	0.245	0.463-3.527	0.636			
Hemoglobin < 110g/L	0.297	0.264-2.092	0.574			
ALT > 50U/L	0.433	0.229-1.840	0.415			
AST > 50U/L	0.356	0.239-2.058	0.581			
Albumin < 32 g/L	1.094	0.842-10.500	0.090			
Total bilirubin < 32 mmol/L	0.009	0.315-3.114	0.987			
Sensitive troponin > 26 ng/ml	0.541	0.226-13.06	0.602			
LDH > 225 U/L	0.725	0.748-5.705	0.162			
Potassium < 3.3mmol/L	0.363	0.519-3.976	0.485			
Soudium < 135mmol/L	1.383	0.894-17.70	0.070			
Prothrombin time > 14.5 seconds	0.052	0.268-3.367	0.936			
APTT > 42 seconds	0.374	0.786-9.927	0.112			
D-dimer > 0.5μg/mL	0.072	0.488-4.826	0.464			
Fibrinogen > 4g/l	0.5	0.597-4.555	0.335			
hsCRP > 30mg/L	1.801	1.701-21.53	0.005*	1.670	0.481-5.802	0.42
IL-6 > 100pg/ml	1.509	1.528-13.384	0.006*	4.938	1.262-19.314	0.022*
IL-4 > 3.2pg/ml	2.144	1.122-644.948	0.038*	2.243	0.225-22.396	0.491
IL-10 > 5pg/ml	0.860	0.839-6.65	0.104			
TNF-α > 23pg/ml	0.551	0.388-7.577	0.471			
IFN-γ > 4pg/ml	1.606	1.123-22.129	0.035*	1.723	0.324-9.158	0.523
CD4+T cells decreased	0.440	0.478-5.048	0.464			
CD8+T cells increased	0.306	0.202-2.680	0.634			

Abbreviation: COVID-19, coronavirus disease 2019; Alanine aminotransferase, ALT; Aspartate aminotransferase, AST; Activated partial thromboplastin time, APTT; High-sensitivity C-reactive protein, hsCRP; Lactate dehydrogenase, LDH; White blood cell, WBC, HR, hazard ratio; CI, confidence interval. Data are expressed as HR and 95% CI. P values mean the comparison between severe COVID-19 with or without comorbidities, *p < 0.05 statistically significant.

## DISCUSSION

The clinical symptoms in COVID-19 patients are similar to those seen during the SARS-CoV outbreak in 2003, but the disease progression in COVID-19 patients is faster. Some patients show atypical respiratory symptoms at the early stage, but can rapidly progress to acute respiratory distress syndrome (ARDS), multi-organ failure, and death in one to two weeks [5]. These symptoms are more evident in severe COVID-19 patients with chronic medical illnesses. Our study confirmed that COVID-19 patients with comorbidities showed more pronounced leukopenia, lymphopenia, hypokalemia, hypoalbuminemia, coagulation disorders and higher levels of inflammatory indices ESR, CRP, IL-6, and IFN-γ. In addition, the overall prognosis for patients with comorbidities was poor, with longer hospital stays and remission duration.

Existing research indicates that the release of pro-inflammatory cytokines *in vivo* may contribute to rapid disease progression. Immune cells release a variety of cytokines, including pro-inflammatory (TNF-α, IFN-γ, IL-2, and IL-6) and anti-inflammatory cytokines (IL-10) to maintain homeostasis in the body [8, 9]. However, infection could induce the massive release of pro-inflammatory factors, which in turn lead to a “cytokine storm” that disrupts immune homeostasis [9]. Studies on SARS-CoV indicate that severe patients show high serum levels of inflammatory factors, such as IFN- γ, IL-1, 2, 6, TNF and TGF-β. These inflammatory factors can induce the apoptosis of vascular endothelial cells and alveolar epithelial cells, increase the permeability of blood vessels, activate macrophages, and recruit neutrophils and fibroblasts. These events subsequently destroy the original immune homeostasis as well as cause substantial damage to lung tissue and other organs, ultimately inducing ARDS, coagulation disorders, and multiple organ failure [8–12].

Compared to the healthy population, patients with comorbidities are more susceptible to COVID-19 as well as prone to rapid progression to severe lesions or death [3, 13]. We found that the serum levels of IL-6, and IFN- γ was positively correlated with the severity of disease. COVID-19 patients coexisting with diabetes, hypertension, or neoplasia had significantly higher levels of IL-6 and IFN-γ from the early stage of infection—usually accompanied by decreased lymphocytes—and were prone to organ and coagulation disorders.

Mounting evidences has confirmed that lymphocytes and lymphocyte subsets are the main protective barrier for cellular and humoral immunity. CD4+ T cells play a key role in regulating CD8+ T cell function, facilitating B-cell responses, and inducing the antibodies that resist virus invasion [14–17]. During the outbreak of SARS and MERS-CoV, more than 80% of patients showed a significant decrease in either CD4+ or CD8+ T cells, which was positively correlated with disease severity [18–20]. In our study, we also found that about 48% of the patients showed a decrease in the number of total lymphocytes, accompanied by a significant decrease in CD4+ T cells subsets. The levels of lymphocyte counts were negatively correlated with inflammatory indices—such as IL-6 and IFN-γ—as well as disease severity, which was more prominent in COVID-19 patients with comorbidities.

In this study we reported 2 deaths, all found in the comorbidities group. Both of the cases developed progressive respiratory distress, cardiac dysfunction, and severe coagulation dysfunction after one week of treatment, accompanied by a progressive increase in serum levels of IL-6 and IFN-γ, lymphopenia, hypokalemia, hypoproteinemia, and coagulation dysfunction. The patients died from multiple organ failure. These findings suggest that COVID-19 cases combined with comorbidities was more prone to trigger the release of inflammatory factors, which may be a key cause for the rapid disease progression and long remission period. Currently, the use of glucocorticoid therapy to control inflammation invasion and inhibition of cytokine storm is controversial [21, 22]. It has been proposed that glucocorticoid therapy could not change the progress of inflammation and reduce antigen clearance [22]. However, in our study, 23 patients in the comorbidities group accepted glucocorticoid therapy. Relieved symptoms and decreased inflammatory factors were observed after treatment, indicating that the timely introduction of glucocorticoid therapy is essential to alleviate disease development and reduce mortality in patients with coexisting comorbidities. However, application of glucocorticoid therapy also indicates that patients' condition is severe, and glucocorticoid therapy may did not significantly reduced the complete remission time and the hospital stay.

Despite our study being a retrospective observational study with a small sample size, it is the first to investigate the clinical characteristics and immune function of severe COVID-19 patients with underlying diseases. We identified that the excessive release of inflammatory factors, especially IL-6, was the internal cause of disease progression in patients. Thus, the timely introduction of anti-inflammatory treatment, such as glucocorticoid therapy, may be necessary.

## MATERIALS AND METHODS

### Patient and study design

This study was a single-center retrospective clinical study and was approved by the Ethics Committee of Huazhong University of Science and Technology. Verbal consent was obtained for urgent data collection. The study population included 79 subjects consecutively admitted to Cancer Center of Union Hospital in Wuhan from February 10 to March 22, 2020. All the patients had laboratory-confirmed cases of COVID-19. Patient characteristics, clinical history, physical examination, laboratory investigations, radiologic findings, and response to treatment were collected and reviewed retrospectively. Subjects were included in this study based on the following eligibility criteria: Age range was 19-80 years (male/female), the patient was a confirmed COVID-19 case based on the CT imaging features, respiratory samples (throat swab/ nasopharyngeal swab/ endotracheal aspirates/ bronchoalveolar lavage) were positive for the RNA virus nucleic acid detection by real-time RT-PCR, or viral genome sequencing revealed a strong homology to COVID-19. Additionally, the clinical manifestations presented with severe pneumonia: fast breathing (respiratory rate ≥ 30/min), dyspnea, lip cyanosis; finger pulse oxygen saturation < 93% at rest; partial pressure of arterial oxygen pressure (PaO_2_, mmHg) / fraction of inspired oxygen (FIO_2_) ratios ≤ 300mmHg (1 mmHg = 0.133 kPa); the CT images showed multiple lesions in bilateral lungs or pulmonary lesions progression > 50% within 24-48 h [7]. Complete CT imaging data before, during, and after antiviral therapy, as well as relevant laboratory examinations were available for all enrolled patients. Follow-up was completed in all enrolled patients.

### Inflammatory factors and lymphocyte subsets analysis

Peripheral blood samples of all enrolled patients were collected and transported to the Immune Research Laboratories, Union Hospital, Huazhong University of Science and Technology. Serum levels of the following cytokines were determined: IL-2R, IL-4, IL-6, IL-10, IFN-γ and TNF-α. Levels of T lymphocyte subsets (CD3+ T cells, CD4+ T cells, and CD8+ T cells) were measured by flow cytometry. Each comparison was computed for all clinical data collected.

### Treatment regimen and efficacy studies

All enrolled patients received standard treatment according to the guidelines for diagnosis and treatment of COVID-19 issued by the 7^th^ edition of National Health Commission of the People’s Republic of China (NHCPRC). The clinical presentations, CT re-examination results, laboratory investigations (routine blood, serum biomarker indicators, total lymphocyte count, CD4+ and CD8+ T cells), inflammatory factors (IL-2R, IL-4, IL-6, IL-10, IFN-γ and TNF-α), Erythrocyte Sedimentation Rate (ESR), and C-reactive protein (CRP) levels were recorded before and every 7-10 days following treatment. Contrast analyses were carried out to evaluate the disease outcome. All patients were followed-up until the disease resolved or death occurred.

### Study endpoint and outcome

The primary outcome was the disease remission time, defined as the time from the onset of treatment to the negative detection of COVID-19, and CT images of remission. The time for negative coronavirus nucleic acid detection (time from onset treatment to negative nucleic acid detection of coronavirus), and time to CT images remission (time from onset treatment to CT images showing the absorption of lung lesions > 20%) were analyzed. Lymphocyte subsets, inflammatory factors analysis, and their correlation with treatment efficacy were set as a secondary outcome.

### Statistical analysis

All data were subjected to statistical analysis with SPSS version 17.0 statistical software and the results were presented as mean ± standard deviation (SD). Student’s t test was used to compare continuous variables and the Chi-squared test was performed to test for between-group differences among the categorical variables. Where the primary end point of this study was disease remission time, the Kaplan-Meier method and Log-rank test were performed for related data representation, comparison, and analysis. Cox regression was used for multivariate analyses to assess the relative risk for each factor including the clinical presentations, routine blood, serum biomarker indicators, inflammatory factors, etc. *P* < 0.05 was regarded as statistically significant.

### Ethical approval and informed consent

This study was a retrospective observational study approved by the Ethics Committee of Huazhong University of Science and Technology, China. The ethical approval number was 0255 [2020].
